# Cellulose from the banana stem: optimization of extraction by response surface methodology (RSM) and charaterization

**DOI:** 10.1016/j.heliyon.2022.e11845

**Published:** 2022-12-05

**Authors:** Nguyen Thi Thuy Van, Pag-asa Gaspillo, Ho Gia Thien Thanh, Nguyen Huynh Thao Nhi, Huynh Nhat Long, Nguyen Tri, Nguyen Thi Truc Van, Tien-Thanh Nguyen, Huynh Ky Phuong Ha

**Affiliations:** a Ho Chi Minh City University of Technology (HCMUT), 268 Ly Thuong Kiet Str., Dist. 10, Ho Chi Minh City, Viet Nam; b Vietnam National University Ho Chi Minh City, Linh Trung Ward, Thu Duc Dist., Ho Chi Minh City, Viet Nam; c Institute of Chemical Technology, Vietnam Academy of Science and Technology, No.1A, TL29 Str., Thanh Loc Ward, Dist. 12, Ho Chi Minh City, Viet Nam; d Department of Chemical Engineering, De La Salle University, Manila, Philippines; e Institute of Drug Quality Control Ho Chi Minh City, 200 Co Bac Str., District 1, Ho Chi Minh City, Viet Nam; f Institute of Materials Science − VAST, 18 Hoang Quoc Viet, Cau Giay District, Hanoi, Viet Nam

**Keywords:** Banana, Cellulose, Modeling and optimization, Response surface methodology

## Abstract

Cellulose was extracted from the banana stem by chemical method and the factors affecting the extraction process such as concentration of NaOH and H_2_O_2_, as well as the assisted microwave time were investigated. Design-Expert software with Response Surface Methodology was used in the modeling and optimization of the cellulose extraction process. The results of XRD, FT-IR, SEM were also used to determine the physicochemical properties of cellulose obtained from the banana stem. The results of the modeling and optimization process of cellulose extraction showed the efficiency of the process and the high applicability of cellulose from the banana stem to create valuable industrial products.

## Highlights

• Microwave-assited chemical extraction of cellulose from the banana stem has been carried out.
• Response Surface Methodology was used in the modeling and optimization of the cellulose extraction.
• Physico-chemical properties of cellulose was assessed.

## 1 Introduction

Recently, the extraction of cellulose from agricultural wastes to produce green and clean products has received great attention in the world. Aerogels prepared using cellulose as precursor own the renewability, biocompatibility, non-toxicity, and biodegradability of cellulose, while maintaining the aforementioned properties. Cellulosic aerogels known as innovative and third generation material within the aerogel family (traditionally silica aerogel and polymer aerogel). The material is versatile in various applications such as optoelectronics, adsorption catalysis, sound and heat insulation, medical materials, aerospace materials, and many other fields [1, 2, 3, 4]. There have been many publishes on the extraction and evaluation of the quality and efficiency of the extraction process from many different materials such as rice straw, corn, sugarcane bagasse, oil palm, starch [5]. Among these raw materials, the banana stem was evaluated as a potential source of cellulose [6, 7, 8, 9, 10]. Banana is planted in many areas in the world and it is one of main export product of many countries. Each hectare of bananas generates about 220 tons of waste, and makes the problem on landfills and polluting the environment [10]. Therefore, making full use of waste from banana trees to create valuable products plays an important role in improving economic efficiency and moving towards sustainable development for banana cultivation as well in treatment of agricultural waste in general.

Cellulose is the main component of the banana stem, it takes about more than 50% of the total banana stem, and this is a natural, renewable, and biodegradable polymer that can be used in many fields such as raw materials for construction, paper, insulation materials, adsorption materials, environmental treatment [8]. Furthermore, several chemicals, including as acids, alkali compounds, and sodium hypochlorite, should be utilized in the extraction process for hydrolysis and raw material bleaching [11]. Previous study has found that alkali treatment employing microwave technology in conjunction with diluted alkali may remove more lignin and hemicellulose from biomass in less time than standard heating. Microwave aided compound extraction from natural plants was researched and proven to have a large increase in extraction yield with shorter extraction time, fewer chemicals employed, and considerable energy savings [12, 13]. It demonstrated quick, homogeneous, and selective heating for chemical change throughout the extraction process [14]. Since then, the usage of chemicals has been reduced. For example, pretreatment of Bermuda grass with 1% NaOH in a microwave at a power level of 250 W for 10 min eliminated about 65% of the lignin while retaining 87% of the glucan [15]. Another research found that the best pretreatment condition was 3% (w/v) NaOH at 180 W for 12 min, with lignin and holocellulose component losses of around 74% and 24.5%, respectively [16]. Thus, microwave irradiation has been shown to improve the saponification of intermolecular ester bonds cross-linking xylan hemicelluloses and other components such as lignin and other hemicelluloses. In general, no studies utilizing microwave aided extraction of cellulose from banana stems have been published previously [17, 18, 19]. To achieve high extraction efficiency while minimizing waste to the environment, the extraction process must be optimized. Modeling and optimization of cellulose extraction from wheat straw, maize cob, palm tree, and orange peel have been studied [20, 21, 22, 23]. However, modeling and optimization of the cellulose extraction process from the banana stem aided microwave have not been described.

Optimizing factorial variable settings such that the response reaches a specified maximum or minimum value is part of the Response Surface Methodology (RSM). Factorial methods like ANOVA are effectively expanded for more in-depth modeling of the effects, but they still serve to model the response. RSM is based on the findings of factorial studies as well as extra treatments that were applied both within and outside of the factorial space (center point and star points). This kind of structure is referred to as a central composite design. Multiple regression analysis yields the upgraded model, and the response surface may be shown using the equation. Plots indicate locations where a reaction exhibits the same magnitude as well as the factors' optimal levels. RSM is appropriate for modeling and optimizing the cellulose extraction process from the banana stem.

In this work, cellulose was extracted from the banana stem using a microwave aid, which improved the cellulose extraction yield. The Response Surface Methodology (RSM) was used, with the input affecting parameters being NaOH and H_2_O_2_ concentrations, and also microwave aided radiation timings. The obtained results of the cellulose, hemicellulose, and lignin contents from the banana stem constitute the process's reaction function. According to the findings, there is a large potential for extracting and using cellulose from banana stem to create green products such as adsorbent, soundproof, and heat-insulating materials, as well as many other disciplines.

## 2 Methodology

### 2.1 Extraction of cellulose

After being washed, the banana stem is cut into small pieces about 1mm in size and dried under sunlight for 8 h. Then, the raw materials will be treated with NaOH solution (with the content of 1, 2, and 3% w/w) with the assistant of microwave (10, 20, and 30 min). The mixture is then removed from the microwave oven and H_2_O_2_ is further added (with a content of 13, 15, and 17% w/w). The mixture is continuing magnetic stirred for 1 h at room temperature with pH of about 11.5. Then, the mixture is filted and washed with distilled water and dried at 70 °C for 24 h and cellulose will be obtained.

### 2.2 Modeling and optimization using RSM

To investigate the effects of different parameters of the cellulose extraction process vis-à-vis the contents of cellulose, hemicellulose and lignin, a Central Composite experimental design was formulated aid by the Design-Expert software version 11.1.0.1 with the 2^3^ factorials. The parameters are considered including the NaOH concentration (1, 2, and 3%), H_2_O_2_ concentration (13, 15, and 17%), and the microwave radiation time (10, 20, and 30 min). In this study, data and results will be analyzed using the Response Surface Methodology (RSM). With this method, the values of the experimental variables are transformed into code variables and are represented as -1, 0, and +1 depending on its relative distance from the central value point. Table 1 is the summary of the transformation of the experimental variables into code variables. The experimental variable of NaOH and H_2_O_2_ concentrations and microwave radiation time were transformed into code variable x_i_ by Eq. (1):

(1) $$x_{i}=\frac{OV-\bar{V}}{\Delta }$$

where: OV is the original variables, *V* is the average of highest and lowest values of the variables and Δ is the average value of the difference between highest and lowest values. The central values were determined at 2% of NaOH, 15% of H_2_O_2_, and 20 min of microwave radiation time.

**Table 1:** Code and Original variables in 2^3^ factorials design.

Parameters	Relation of code and original variables		
	-1	0	1
Concentration of NaOH, C_A_ (%)	1	2	3
Concentration of H_2_O_2_, C_P_ (%)	13	15	17
Microwave radiation time, t (min)	10	20	30

While, Table 2 shows the design matrix of responses expressed as the percentage of cellulose (Y_C_), hemicellulose (Y_H_) and lignin (Y_L_) after the treatment process. The code variables used are X_1_ for NaOH concentration, X_2_ for H_2_O_2_ concentration, and X_3_ for microwave radiation time. In this table, Ci (with i from 1 to 4) represents experimental runs at the center of the model, xi = 0.

**Table 2:** The 2^3^ factorials design with 4 center points including the corresponding responses.

No. of experiments	X_1_	X_2_	X_3_	C_A_ (%)	t (mins)	C_P_ (%)	Y_C_ (%)	Y_H_ (%)	Y_L_ (%)
1	-1	-1	-1	1	10	13	74.91	25.05	3.14
2	1	-1	-1	3	10	13	75.65	24.35	2.63
3	-1	1	-1	1	30	13	76.99	23.05	2.53
4	1	1	-1	3	30	13	79.16	20.85	2.46
5	-1	-1	1	1	10	17	78.15	21.84	2.52
6	1	-1	1	3	10	17	78.45	21.58	2.42
7	-1	1	1	1	30	17	77.42	22.56	3.35
8	1	1	1	3	30	17	79.43	20.52	3.53
9	-α	0	0	0.32	20	15	76.54	23.45	3.15
10	+α	0	0	3.68	20	15	79.02	20.99	2.85
11	0	-α	0	2	3.20	15	77.75	22.24	1.94
12	0	+α	0	2	36.80	15	80.21	19.82	2.42
13	0	0	-α	2	20	11.64	75.86	24.1	2.92
14	0	0	+α	2	20	18.36	78.99	21.03	3.35
C1	0	0	0	2	20	15	82.15	17.89	2.16
C2	0	0	0	2	20	15	81.17	18.86	2.19
C3	0	0	0	2	20	15	82.21	17.81	2.16
C4	0	0	0	2	20	15	82.17	17.84	2.23

### 2.3 Characteristics of samples

The chemical compositions including cellulose, lignin, hemicellulose of raw material (dried banana stem) and the products obtained after each extraction process were analysed to determine following standard TAPPI protocols (α-Cellulose – TAPPI method with TAPPI T203-99; holocellulose with TAPPI T249-75, and lignin with TAPPI T222-88, whilst the hemicellulose was determined by deduction with α-cellulose from holocellulose.

**Figure 1:**
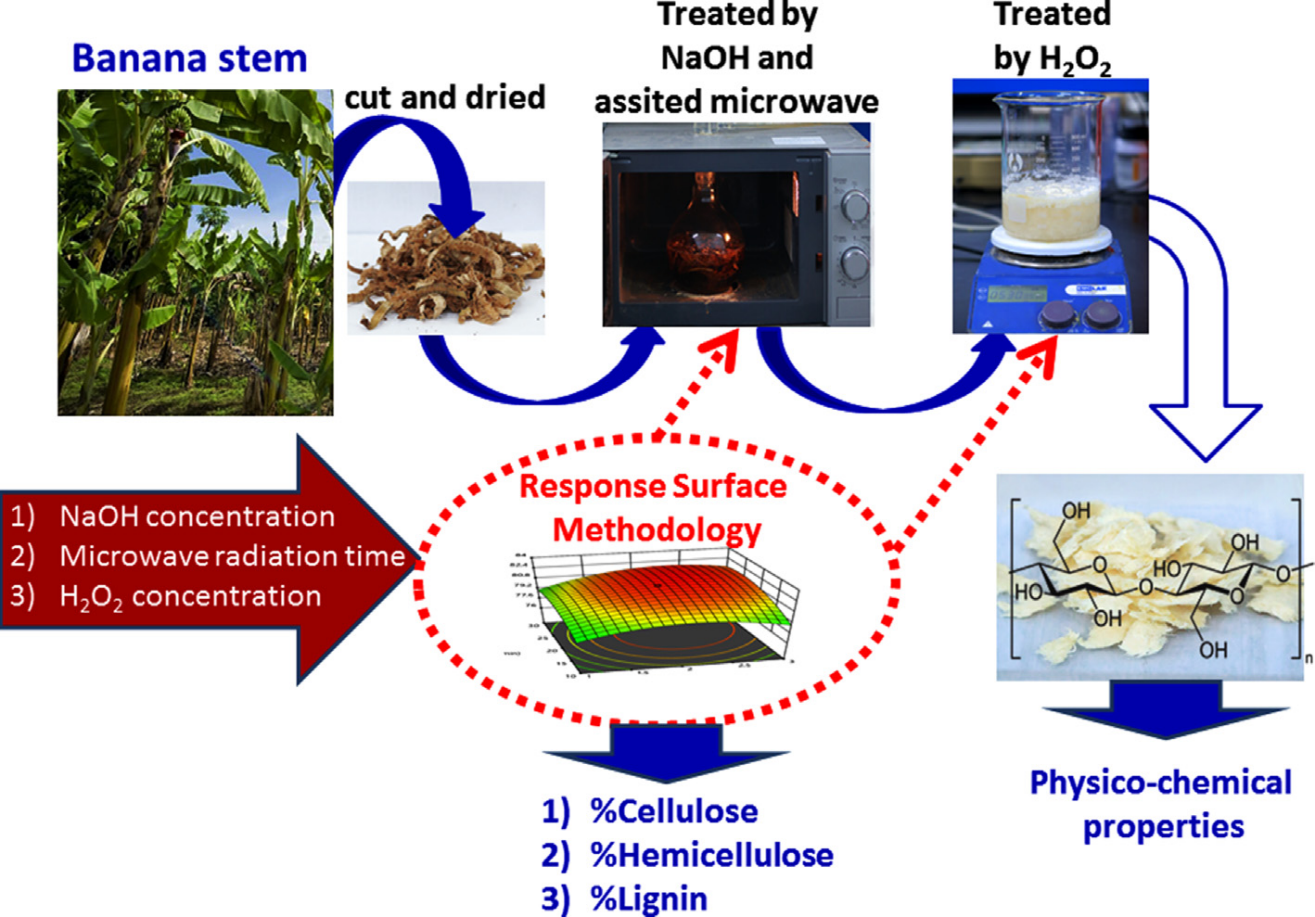
Schematic illustration of the experimental process.

The obtained cellulose sample from the extraction process was characterized with Fourier transform infrared spectra (FT-IR Tensor 27, Bruker), the crystalline structures (XRD, Bruker D8 Advance diffractometer) and FE-SEM field emission scanning electron microscope (FE-SEM, Hitachi, S-4800). Fabrication and characterization of sample are illustrated in Figure 1.

## 3 Results and discussion

### 3.1 Modeling and numerical optimization of the extraction process

The obtained yield of cellulose from banana stem the main purpose of the chemical extraction process. Table 3 of Sequential Model Sum of Squares [Type I] was performed for the selected model for cellulose extraction yield (Y_C_). It can be observed that a Quadratic vs 2FI model was chosen based on the probability value (p-value) of the various models under consideration (<0.0001).

**Table 3:** Sequential model sum of squares [Type I].

Source	Sum of Squares	df	Mean Square	F-value	p-value	
Mean vs Total	1.114E+05	1	1.114E+05			
Linear vs Mean	24.30	3	8.10	1.79	0.1960	
2FI vs Linear	4.84	3	1.61	0.3027	0.8229	
Quadratic vs 2FI	57.53	3	19.18	136.67	<0.0001	Suggested
Cubic vs Quadratic	0.0593	4	0.0148	0.0558	0.9919	Aliased
Residual	1.06	4	0.2658			
Total	1.115E+05	18	6195.33			

Select the highest order polynomial where the additional terms are significant, and the model is not aliased.

Table 4 shows the summary of results when analysis of variance (ANOVA) was performed for selected models. The coefficient in this table was accepted with value is significant when the probability value (p-value) is below 0.05. In this case A, B, C, AB, BC, A^2^, B^2^, C^2^ are significant model terms. Values greater than 0.05 indicate the model terms are not significant [24]. If there are many insignificant model terms (not counting those required to support hierarchy), model reduction may improve your model.

**Table 4:** ANOVA for Quadratic vs 2FI model of cellulose extraction.

Source	Sum of Squares	df	Mean Square	F-value	p-value	
Model	86.67	9	9.63	68.63	<0.0001	significant
A (for C_A_)	6.46	1	6.46	46.02	0.0001	significant
B (for t)	7.29	1	7.29	51.95	<0.0001	significant
C (for C_P_)	10.55	1	10.55	75.19	<0.0001	significant
AB	1.23	1	1.23	8.78	0.0180	significant
AC	0.0450	1	0.0450	0.3207	0.5867	nonsignificant
BC	3.56	1	3.56	25.40	0.0010	significant
A^2^	29.69	1	29.69	211.58	<0.0001	significant
B^2^	15.52	1	15.52	110.62	<0.0001	significant
C^2^	34.75	1	34.75	247.66	<0.0001	significant
Std. Dev.	0.3746	R^2^		0.9872		
Mean	78.68	Adjusted R^2^		0.9728		
C.V. %	0.4761	Predicted R^2^		0.9534		

The Predicted R^2^ of 0.9534 is in reasonable agreement with the Adjusted R^2^ of 0.9728 shows that the difference in this model is less than 0.2. It could be gleaned from this result that the percentage of cellulose extracted from banana waste (R1) can best be represented by the model expressed in terms of code variables as Eq. (2):

(2) R1 = 81.9399 + 0.687629X_1_ + 0.730564X_2_ + 0.878973X_3_ + 0.3925X_1_X_2_ - 0.6675X_2_X_3_ -1.53202 (X_1_)^2^–1.10775(X_2_)^2^–1.65753.(X_3_)^2^

where X_1_, X_2_, and X_3_ are the code variables of NaOH concentration, the H_2_O_2_ concentration, and the microwave radiation time, respectively.

The equation in terms of actual factors can be used to make predictions about the response for given levels of each factor. Here, the levels should be specified in the original units for each factor and the Final Equation in Terms of Actual Factors can be represented as Eq. (3):

(3) R1 = -40.8515 + 6.5932×C_A_ + 0.938283×t + 13.6135×C_P_ + 0.03925×C_A_×t - 0.033375×t×C_P_ - 1.53202×C_A_^2^ - 0.0110775×t^2^ - 0.414383×C_P_^2^

where C_A_, t, and Cp are the NaOH concentration, the microwave assisted time, and the H_2_O_2_ concentration (listed in Table 1).

It should be note that the factor X_1_X_3_ was rejected because it's p-values is not significant. It can be explained that NaOH was added first and then H_2_O_2_, so the effect of two these steps are separately, while the microwave assisted time was effected on both steps.

**Figure 2:**
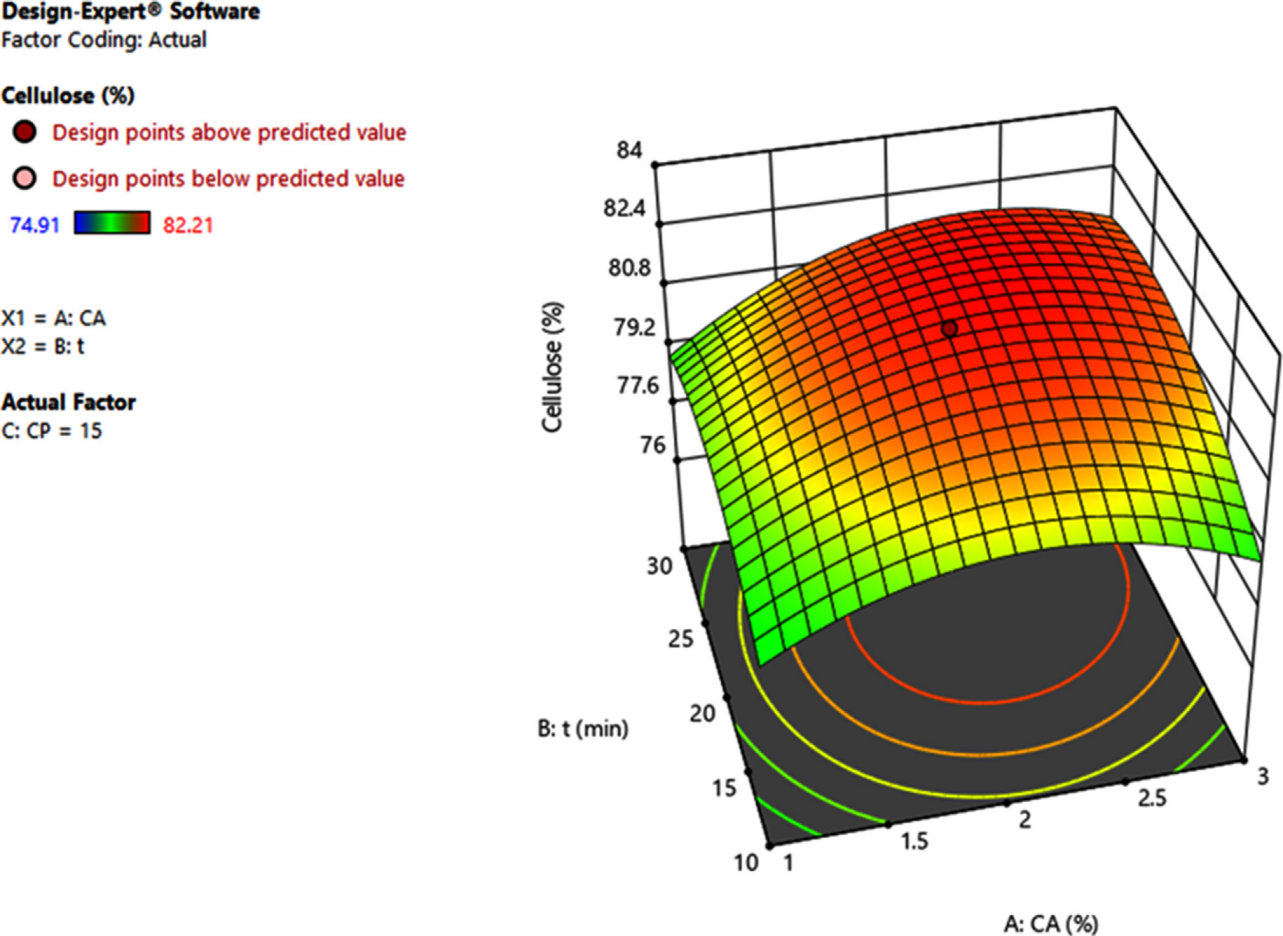
3D graphical representations of the model of cellulose percentage against the concentration of NaOH (C_A_) and microwave assisted time (t).

**Figure 3:**
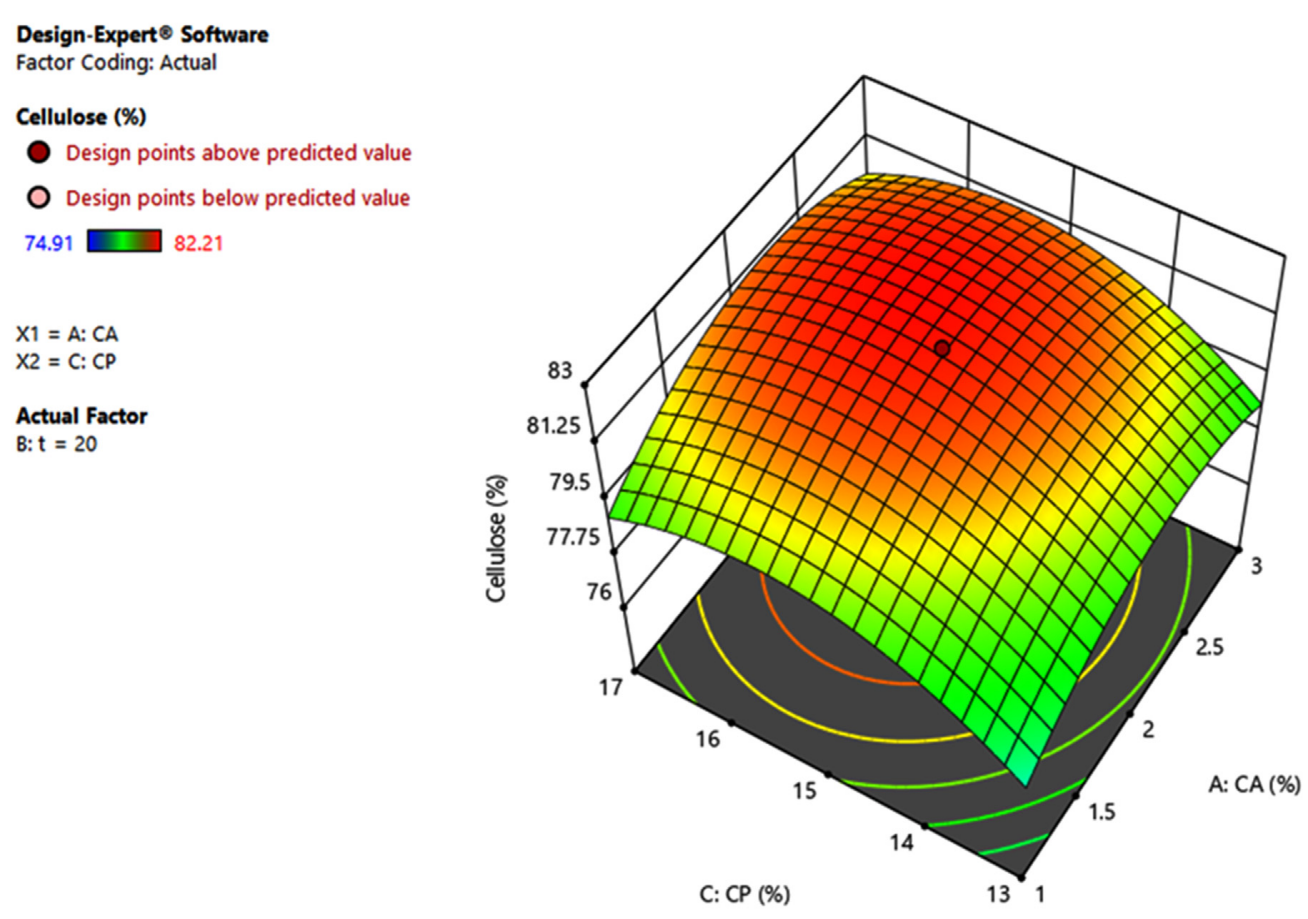
3D graphical representations of the model of cellulose percentage against the concentration of NaOH (C_A_) and H_2_O_2_ (C_P_).

**Figure 4:**
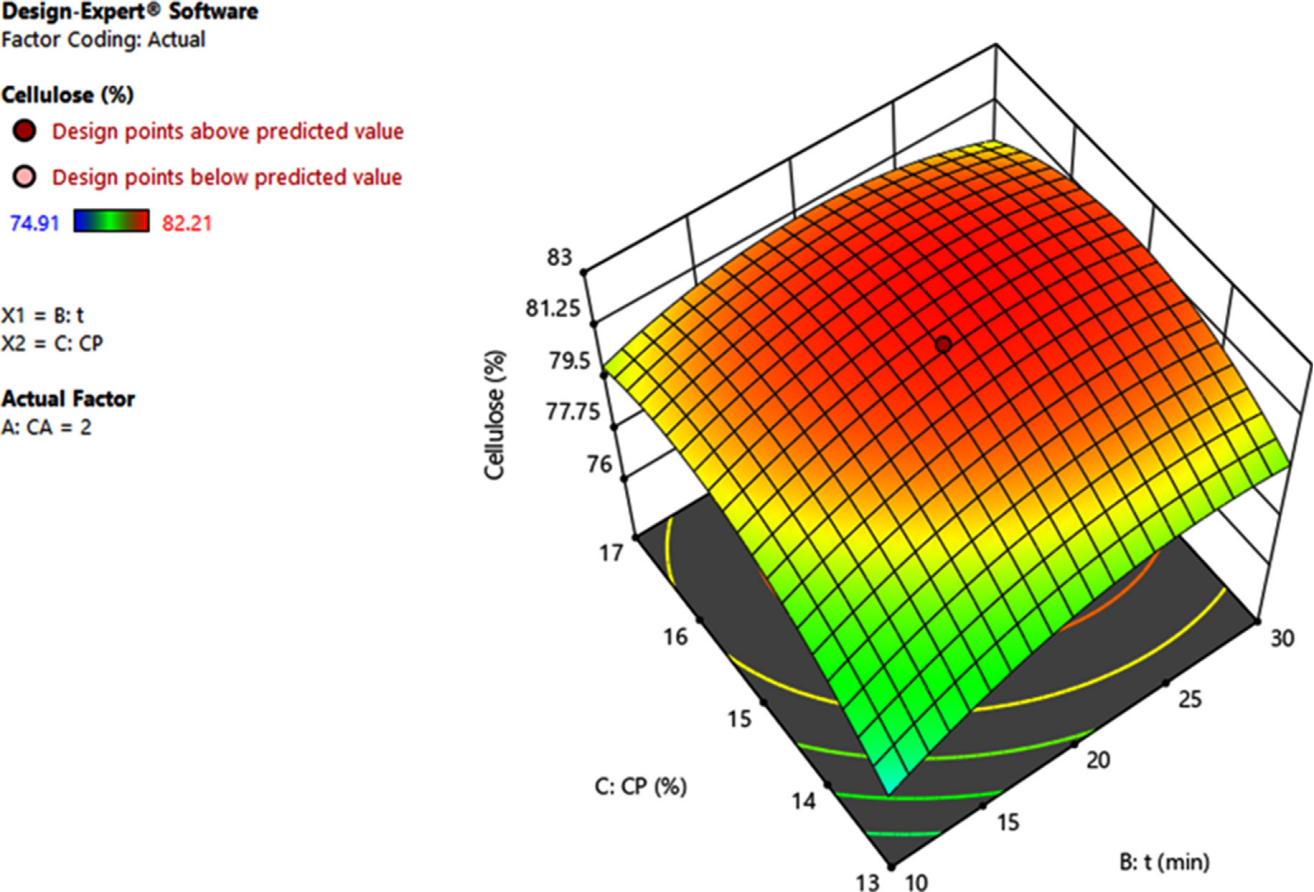
3D graphical representations of the model of cellulose percentage against the concentration of H_2_O_2_ (C_P_) and microwave assisted time (t).

Shown on Figures 2, 3, and 4 are the 3D graphical representations of the model of cellulose percentage against the concentration of NaOH (C_A_), H_2_O_2_ (C_P_), and microwave assisted time (t).

Based on the results obtained from these graphs, it can be found that increasing the content of NaOH and H_2_O_2_ will lead to an increase in cellulose content recovery. This could be explained by the fact that alkaline hydrolysis reduces the amount of hemicellulose and lignin by destroying the bonds between the aryl-ether, carbon-carbon, aryl-aryl functional groups and the ester groups between lignin and hemicellulose [9, 25, 26]. But if NaOH is increased too much will lead to uncontrolled hydrolysis of functional groups in cellulose, then cellulose content in product will be reduced. Similarly, H_2_O_2_ is a strong oxidizing compound that can be used to remove hemicellulose and lignin in alkaline media, but if H_2_O_2_ is used with high concentration will cause cellulose to also decompose [9, 27, 28]. Increasing the MW assisted time also positively affects the cellulose recovery, but increasing the time too long causes the cellulose to degrade, leading to a decrease in cellulose recovery [9].

Similarly, the results of modeling process of hemicellulose and lignin with the data in Table 2 using RSM are also obtained, and the ANOVA tables are shown in Tables 5 and 6.

**Table 5:** ANOVA for Quadratic vs 2FI model of hemicellulose extraction.

Source	Sum of Squares	df	Mean Square	F-value	p-value	
Model	85.51	9	9.50	68.29	<0.0001	significant
A-A	6.38	1	6.38	45.88	0.0001	significant
B–B	7.19	1	7.19	51.68	<0.0001	significant
C–C	10.48	1	10.48	75.32	<0.0001	significant
AB	1.34	1	1.34	9.67	0.0145	significant
AC	0.0450	1	0.0450	0.3234	0.5852	nonsignificant
BC	3.33	1	3.33	23.92	0.0012	significant
A^2^	29.32	1	29.32	210.72	<0.0001	significant
B^2^	15.35	1	15.35	110.34	<0.0001	significant
C^2^	34.20	1	34.20	245.83	<0.0001	significant
Std. Dev.	0.3730	R^2^	0.9872			
Mean	21.32	Adjusted R^2^	0.9727			
C.V. %	1.75	Predicted R^2^	0.9544			

The Predicted R^2^ of 0.9544 is in reasonable agreement with the Adjusted R^2^ of 0.9727; i.e. the difference is less than 0.2.

**Table 6:** ANOVA for Quadratic vs 2FI model of lignin extraction.

Source	Sum of Squares	df	Mean Square	F-value	p-value	
Model	3.89	9	0.4322	356.79	<0.0001	significant
A-A	0.0739	1	0.0739	60.99	<0.0001	significant
B–B	0.2834	1	0.2834	233.92	<0.0001	significant
C–C	0.2328	1	0.2328	192.19	<0.0001	significant
AB	0.0648	1	0.0648	53.49	<0.0001	significant
AC	0.0545	1	0.0545	44.95	0.0002	significant
BC	0.9248	1	0.9248	763.38	<0.0001	significant
A^2^	1.07	1	1.07	886.89	<0.0001	significant
B^2^	0.0000	1	0.0000	0.0245	0.8794	nonsignificant
C^2^	1.46	1	1.46	1201.17	<0.0001	significant
Std. Dev.	0.0348	R^2^			0.9975	
Mean	2.66	Adjusted R^2^			0.9947	
C.V. %	1.31	Predicted R^2^			0.9848	

The Predicted R^2^ of 0.9848 is in reasonable agreement with the Adjusted R^2^ of 0.9947; i.e. the difference is less than 0.2.

The equation in terms of actual factors in the case of hemicellulose content (R2) can be presented as Eq. (4):

(4) R2 = 139.628–6.5159×C_A_ - 0.914993×t - 13.491×C_P_ - 0.041×C_A_×t + 0.03225×t×C_P_ + 1.52243×C_A_^2^ + 0.011017×t^2^ + 0.4111×C_P_^2^

where C_A_, t, and Cp are the NaOH concentration, the microwave assisted time, and the H_2_O_2_ concentration.

In this equation, the coefficient of C_A_×C_P_ is also not significant as obtained from ANOVA result, and showed that the effect of two concentrations of NaOH and H_2_O_2_ combination is not strong as in the case of cellulose. But in the case of lignin (R3), the equation in terms of actual factors (Eq. (5)) showed that coefficient of C_A_×C_P_ is significant because the oxidation of H_2_O_2_ is increased in alkali medium so the decomposition of lignin is effected strongly on the concentration of these two compound [9, 28].

(5) R3 = 28.0121–2.03809×C_A_ - 0.259208×t - 2.90104×C_P_ + 0.009×C_A_×t + 0.04125×C_A_×C_P_ + 0.017×t×C_P_ + 0.291447×C_A_^2^ + 0.0847942×C_P_^2^

where C_A_, t, and Cp are the NaOH concentration, the microwave assisted time, and the H_2_O_2_ concentration.

In optimizing of the cellulose extraction process the concentration of NaOH, H_2_O_2_ and MW assisted time are set “in ranged”. The cellulose content is set as “maximize”, while the hemicellulose and lignin content are set of responses that need to be “minimized”, as shown in Table 7. It exhibits the desire combinations of process parameters that would provide the highest responses of cellulose content and lowest content of hemicellulose and lignin contents.

**Table 7:** Set goal for factors and responses for the Optimization of formulation.

Name	Goal	Lower Limit	Upper Limit	Lower Weight	Upper Weight	Importance
A:C_A_	is in range	1	3	1	1	3
B:t	is in range	10	30	1	1	3
C:C_P_	is in range	13	17	1	1	3
Cellulose	maximize	74.91	82.21	1	1	3
Hemicellulose	minimize	17.81	25.05	1	1	3
Lignin	minimize	1.94	3.53	1	1	3

Optimizing the process conditions with the RSM revealed that the optimum conditions are shown in Table 8. The results show that with the MW assisted time is 21.29 min, and the concentrations of NaOH and H_2_O_2_ are 2.91% and 15.10%, respectively, the highest content of cellulose will be obtained with the value of 82.14%. While the lowest obtained content of hemicellulose and lignin are 17.89% and 2.21%, respectively.

**Table 8:** The result of the optimization formulation of the cellulose extraction process.

Factors/Responses	Values	Desirability	Result
CA (NaOH concentration, %)	2.91	0.933	Selected
T (MW assisted time, min)	21.29		
CP (H_2_O_2_ concentration, %)	15.10		
Cellulose content (%)	82.14		
Hemicellulose content (%)	17.89		
Lignin content (%)	2.21		

According to the results in Table 9, the use of cellulose extraction methods from banana stem with microwave heating is more efficient than conventional extraction methods, according to the results in Table 9, notably in terms of heating time and temperature. Furthermore, our analysis confirms the banana stem's high cellulose and low lignin concentration, validating its promise as a viable source of pulp. The variation in composition is also related to species, geographical location, age, and climatic circumstances, as previously reported [32, 33]. As be known, microwave-assisted extraction (MAE) was applied in this work as an innovative approach for ecologically friendly simple, applying electromagnetic radiation to a material [34]. The results of this approach shown that the cellulose extraction process may be completed in a short period of time [35]. MAE is an exciting technique since it can speed up the process of isolating biomaterials. It is also more convenient to use an environmentally benign hydrogen peroxide (H_2_O_2_) bleaching agent instead of NaClO_2_ [36]. Internal heat is produced via microwave heating, in which microwave radiation directly interacts with molecules, creating a fast increase in temperature due to dipole rotation and ion conduction [37]. The microwave heating method relies on collisions between polar molecules to transform kinetic energy into heat. The increased power generates an increase in energy and temperature, which breaks the fiber and degrades the lignin [38], resulting in a much greater extraction yield of cellulose from banana stem than previously published data.

**Table 9:** Comparison of cellulose extraction conditions from banana stem by different methods and composition of obtained cellulose product.

Method	Fixed conditions			Composition of product			Refs.
	NaOH (%)	Time (mins)	H_2_O_2_ (%)	L (wt.%)	H (wt.%)	C (wt.%)	
MCP	2.91	21.29	15.10	2.21	17.89	82.14	This work
	4	14	5	-	-	86.43	[29]
HCP	17.7	30	-	15.30	8.80	69.40	[19]
	2	3240	-	17.58 ± 0.40	13.19 ± 1.05	33.86 ± 1.53	[30]
	5	600	-	4	6	90	[31]

Note: Lignin (L), Hemicellulose (H), Cellulose (C), Microwave - assisted chemical pretreatments (MCP), and Heating-assisted chemical pretreatments (HCP).

### 3.2 Cellulose's characteristics

**Figure 5:**
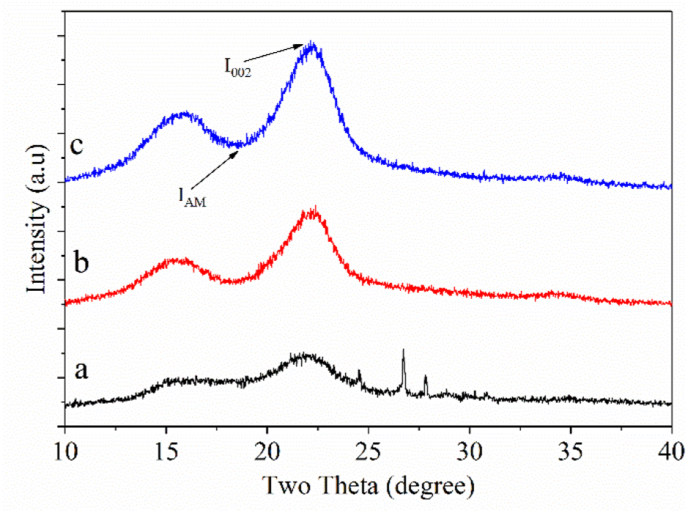
XRD patterns of samples; a) Raw banana stem powder, b) treated with 2.9% NaOH solution for 21.29 min, and c) treated with NaOH 2.91 %w/w for 21.29 min and then with H_2_O_2_ 15.10 %w/w for 1 h.

Figure 5 shows XRD analysis results of raw material and samples after treated with NaOH and H_2_O_2_. The crystallinity index (CrI), crystal thickness, and distance between the lattices were all calculated using the data of the samples presented in Table 8. It can be seen that the pretreatment reduced the amorphous area (2θ angle of 18°) while increasing the crystalline region (2θ angle of 22°). The raw material (line a) has two main diffraction peaks, namely at 2θ = 15° and 2θ = 22.1° with diffraction lattice surfaces of (110) and (200), respectively. In addition, a low-intensity peak is observed at 2θ = 34.4° with a diffraction lattice of (040). This result shows that cellulose from the banana stem has the characteristics of type I cellulose structure [39,40,41]. This crystal structure corresponds to a parallel arrangement of the linear chains of cellulose glycosidic bonds and is known as polymorph cellulose with higher mechanical strength [41]. The crystallization index increased from 57.87% (original raw materials, line a) to 74.20% when preliminary processing with NaOH (line b) and 82.78% when pre-processed with NaOH and H2O2 (line c). This result demonstrates that the CrI of the sample is influenced by the chemical components in the sample, in which the composition of the banana stem contains only crystalline cellulose. Thus, the free hydroxyl groups present in cellulose macromolecules are likely to have participated in several intramolecular hydrogen bonds giving rise to different crystal arrangements. Therefore, after treatment with NaOH and H_2_O_2_ - two amorphous substances are lignin and hemicellulose of the banana stem were removed leading to an increase in CrI [42]. Although in the pretreatment of raw materials with NaOH and H_2_O_2_, enlargement of hemicellulose leads to a decrease in cellulose crystallization. However, the CrI of the sample after being treated with NaOH with H_2_O_2_ is still higher than that of the raw banana stem due to the amount of more lignin and hemicellulose have been removed than small amounts of crystalline cellulose that were originally about to be converted into microcrystalline cellulose. Table 10 also shows that following treatment with NaOH and both NaOH and H_2_O_2_, crystal thickness increases, and comparable results have been previously reported [42, 43]. After the chemical treatment, the distance between the lattices (d-spacing) is decreased [42].

**Table 10:** Crystallinity index, crystal thickness, and d-spacing before and after chemical treatment.

Samples	Crystallinity index, (CrI, %)	Crystal thickness, nm	d-spacing, nm
Raw	57.87	2.82	0.41
After treated with NaOH	74.20	3.07	0.40
After treated with NaOH and H_2_O_2_	82.78	3.13	0.40

**Figure 6:**
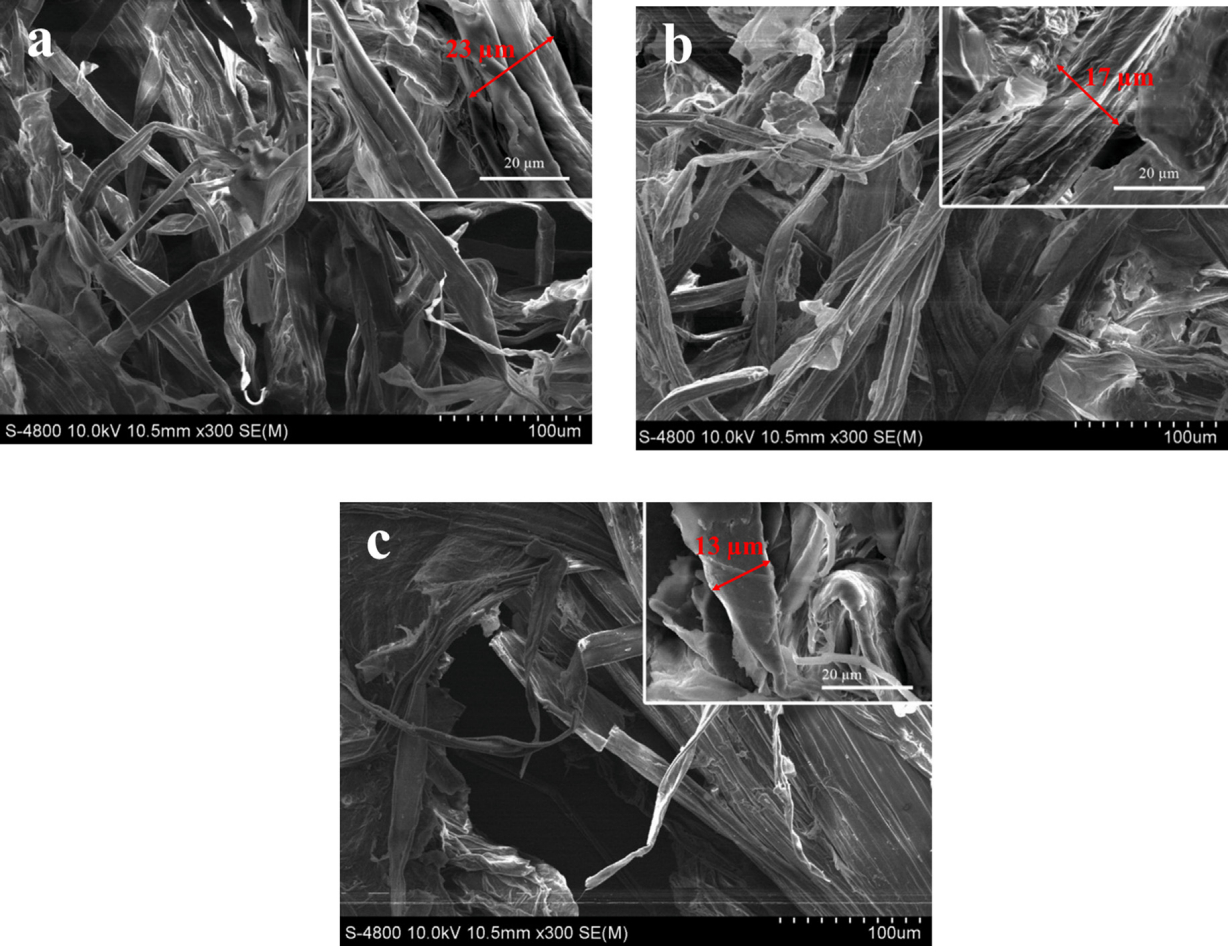
SEM images of samples; a) Raw banana stem powder, b) treated with 2.91% NaOH solution for 21.29 min, and c) treated with NaOH 2.91 %w/w for 21.29 min and then with H_2_O_2_ 15.10 %w/w for 1 h.

From the SEM result of Figure 6a, the microstructure of the raw material surface is observed. The results revealed that the surface of the cellulose fibers from the raw banana stem is relatively smooth, intact, without holes, and arranged in order. This is because the exterior is covered with wax layers, pectin along with other impurities such as hemicellulose, lignin. In addition, these layers also have the function of protecting the inner cellulose [44]. Figure 6b shows the image of the material after being treated with NaOH, it can be clear that the surface of the material has become rough, fragmented, and begins to appear with cracks and holes. The reason for these changes is because of the depletion of hemicellulose and lignin, which leads to the fibers becoming swollen and the bonds of hemicellulose, and lignin broken down, causing the presence of the inner cellulose layers [45]. After being bleached with H_2_O_2_ (Figure 6c), the cellulose fibers were cut apart, the appearance of voids, surface cracks were more obvious, the average diameter of the material was smaller than that of the raw material initially, and after NaOH treatment [3]. This proves that components such as wax, pectin was removed, the bonds of hemicellulose, and lignin are severed due to the swelling of cellulose fibers and the free radical reaction of the hydroxyl group with lignin.

**Figure 7:**
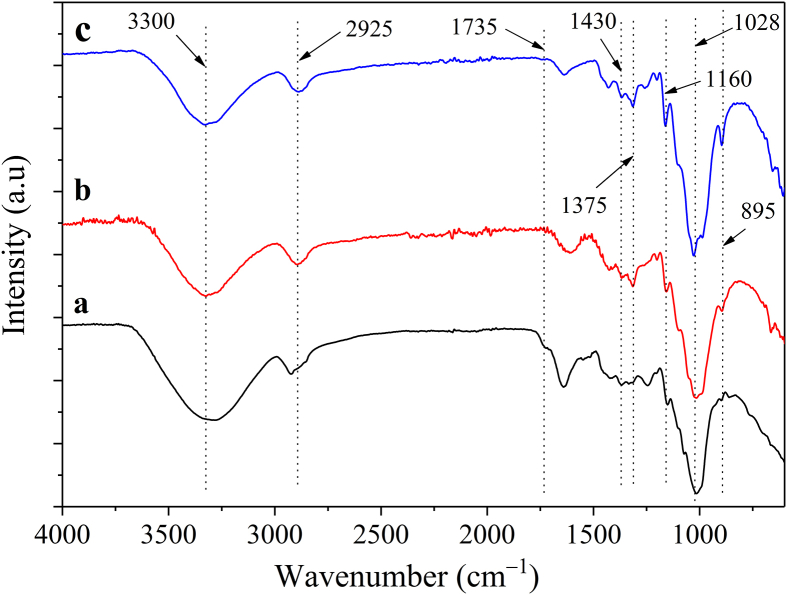
FT-IR spectra of samples; a) Raw banana stem powder, b) treated with 2.91% NaOH solution for 21.29 min, and c) treated with NaOH 2.91 %w/w for 21.29 min and then with H_2_O_2_ 15.10 %w/w for 1 h.

FT-IR infrared spectroscopy can be used to determine the presence of cellulose, hemicellulose, and lignin before and after specific processing. The results in Figure 7 show that there are two major absorption regions, an area with a low wavenumber in the range of 750–1800 cm^−1^ and an area with a higher wavenumber corresponding to about 2700–3500 cm^−1^. The absorption band around 3300 and 2925 cm^−1^ was related to the hydrogen-bonded OH groups and the asymmetric chemotherapy oscillations of the C–H bonds in the –CH_2_ group of cellulose, respectively [46]. The characteristics signals located at 1430 and 1375 cm^−1^ are attributed to the deformation oscillations in and out of the plane of the CH_2_ and CH groups, respectively [47, 48]. Furthermore, the abrupt small peak at 1160 cm^−1^ is associated with C–O–C asymmetrical stretching [49]. The broad sharp peak absorbed in the 1028 cm^−1^ region, as shown in Figure 7, corresponds to the C–O stretching vibration from the cellulose, which increased peak intensity after pectin, hemicellulose, and lignin were removed [47]. Similarly, the existence of a peak at 895 cm^−1^ is linked with C–O groups, which are connected to *β*-glycosidic connections [50, 51]. The signal intensity of these peaks increases sharper after bleaching than the peaks in the coarse banana stem when treated with alkali. Moreover, the shoulder peak at 1735 cm^−1^ is attributed to the carbonyl group of aromatic acids found in lignin and hemicellulose in raw banana stem [52]. Following treatment, the absence of the peak indicating the cleavage of the linkages in the fibers between ferulic acid, *p*-coumaric acid, or (*p*-) hydroxycinnamic acids and lignin resulted in a significant reduction in hemicellulose and lignin content and an increase in cellulose content in the banana stem [53].

## 4 Conclusions

Chemical extraction of cellulose from banana stem has been carried out. RSM was used to model and optimize the influencing factors such as NaOH content, H_2_O_2_ content and microwave extraction time to obtain optimal conditions for extraction. At the NaOH concentration of 2.9%, the concentration of H_2_O_2_ of 15.10% with the extraction time of 21.29 min, the maximum cellulose content obtained was 82.136%, while the hemicellulose and lignin contents obtained were the lowest, respectively 17.8900% and 2.212%. The results obtained from XRD showed that the Crystallinity index as well as the distance between the lattices increased significantly after chemical extraction. The SEM results showed that wax components, pectin were removed, the bonds in hemicellulose and lignin were severed and thus there was a significant increase in porosity. The FT-IR results confirmed that the hemicellulose and lignin content was significantly removed and the cellulose content in the banana stem increased after treatment.

## Declaration

### Author contribution statement

Nguyen Thi Thuy Van, Nguyen Tri, Huynh Ky Phuong Ha: Conceived and designed the experiments; Analyzed and interpreted the data; Wrote the paper.

Pag-asa Gaspillo: Performed the experiments; Analyzed and interpreted the data; Contributed reagents, materials, analysis tools or data.

Ho Gia Thien Thanh: Conceived and designed the experiments; Wrote the paper.

Nguyen Huynh Thao Nhi, Huynh Nhat Long: Conceived and designed the experiments; Contributed reagents, materials, analysis tools or data.

Nguyen Thi Truc Van, Tien-Thanh Nguyen: Contributed reagents, materials, analysis tools or data.

### Funding statement

This work was supported by the program code VAST07.04/22-23 from Vietnam Academy of Science and Technology.

### Data availability statement

Data included in article/supplementary material/referenced in article.

### Declaration of interest's statement

The authors declare no conflict of interest.

### Additional information

No additional information is available for this paper.
